# Comparative analysis of mRNA transcripts of HT-29 cell line expressed in identical quantities for pathogenic *E. coli* strains UM146 and UM147 with control *Escherichia coli* Nissle 1917

**DOI:** 10.7150/jgen.67277

**Published:** 2022-01-01

**Authors:** Roman Kotłowski

**Affiliations:** Department of Molecular Biotechnology and Microbiology, Gdansk University of Technology, Faculty of Chemistry, Narutowicza 11/12, 80-233 Gdansk, Poland.

**Keywords:** HT-29 cell line, *Escherichia coli*, PAI I, trypsin-like activity, infection

## Abstract

Aim of study was comparative analysis of mRNA transcripts of HT-29 cell line, expressed in identical quantities for the combination of pathogenic and non-pathogenic *Escherichia coli* strains. HT-29 confluent monolayers infection with two pathogenic *E. coli* strains UM146 and UM147 resulted in two sets of mRNA transcripts that were identical with RNA transcripts obtained for non-pathogenic one strain *E. coli* Nissle 1917. In this study genome-wide experiments were conducted using expression microarray-system. Only one common mRNA transcript coding for CCDC65 gene was equally expressed by HT-29 cells after incubation challenge with three different *E. coli* strains used. This gene and its bacterial analogue are important in the ciliary or flagellar motility, respectively. Altogether, 78 and 81 HT-29 mRNA transcripts for *E. coli* UM146 and *E. coli* UM147 had identical RNA quantity in comparison to the response obtained for non-pathogenic *E. coli* Nissle 1917 interactions with HT-29 monolayers. Specific analysis using REACTOME and agriGO terms enrichment data-mining tools as well as word-cloud analysis allowed for identification the most important processes characteristic during HT-29 cell line infections for each pathogenic *E. coli* strain used. The importance of results may contribute to recognition of those processes during bacterial infections that are identical with processes arising from human interaction with non-pathogenic strains that belong to the same bacterial species.

## Introduction

The majority of studies concerning host-pathogen interactions are focused on characterization of the pathogenic bacterial strains in confrontation to non-pathogenic control bacterial species. However, in this study different strategy during subtractive experiment in the human-genome wide scale was conducted. The difference was related to identification of only those mRNA transcripts that were expressed in the same quantity also for non-pathogenic bacterial strain used as control. The obtained results of our model studies may help to understand the basis of immunological responses that are constant regardless of invading or existing in commensal manner strains that interact with host cells. Also, some implications can be made for understanding the mechanisms of human infections from variety of bacterial toxins present in two different *E. coli* strains and different H flagellar types and some minor differences in amino-acid sequences of FimH, Mdh and PurA proteins previously reported 1.

*E. coli* isolated from Crohn's Disease patient and *E. coli* strain derived from Ulcerative Colitis patient used in this host-pathogen model study extend the current knowledge about inhibitory effect of *Escherichia coli* strain Nissle 1917 (EcN) on adhesion to and invasion of intestinal epithelial cells during probiotic treatment with EcN 2. Also, *Lactobacillus casei* DN-114 001 3 known by strong inhibitory interaction of adherent-invasive *E. coli* with intestinal epithelial cells confirms the significant role of presented studies in better understanding of bacterial aetiology of Crohn's Disease.

## Materials and methods

### Cell line

HT-29 cell line stored originally in liquid nitrogen was cultured in the RPMI 1640 medium supplemented in 10% with fetal bovine serum until 70% of the bottom line of culture bottles with the surface of 75 cm^2^ were covered by HT-29 cells monolayer. Observation by inverted microscope was conducted to estimate the confluent status of cell line. Subsequently, 3h incubation time of 1 ml bacterial cultures ≈10^7^ CFU followed by pipetting of confluent cell line was conducted at 37 °C at the presence of normal atmosphere supplemented with 5% CO_2_.

### Micro-array

The total RNA was extracted from HT-29 cell line based on Chmczynski and Sacchi protocol following further RNA processing in accordance with the Affymetrix® recommendations for GeneChip HG-U133A Plus 2.0 microarray expression chips protocol. Subsequently, biotinylated RNA was stained in fluidic station and emission of signals for entire human genome was determined using Affymetrix® scanner.

### Bacterial strains

Pathogenic *E. coli* strains UM147 and UM146 have differences identified in amino-acid sequence of Mdh coding for malate dehydrogenase, PurA encoding adenylosuccinate dehydrogenase and FimH protein present in fimbria type I. In addition, *E. coli* UM146 has PAI I pathogenicity island and has H7 type of flagella in contrast to H5-type present in UM147 and H1 present in control strain *E. coli* Nissle 1917. However, unique feature of *E. coli* UM147 is trypsin like activity determined during cell line culture.

### Statistical analysis

Statistical test methods applied in agriGO: GO Analysis Toolkit and Database for Agricultural Community (cau.edu.cn) (http://bioinfo.cau.edu.cn/agriGO/) were as follows: Chi-Square; Multi-test adjustment method Hohberg FDR; Significant level 0.1. In the REACTOME online tool only biochemical reactions that significantly (p<0.05) represented particular biochemical pathway were selected for comparative analysis.

## Results and Discussion

In the present study two sets of RNA transcripts determined for HT-29 genes after infection with two pathogenic *E. coli* strains UM146 and UM147 and one non-pathogenic *E. coli* Nissle 1917. The subtraction criteria relayed on identical quantity of cell-line transcripts response for two separately analyzed pathogenic *E. coli* strains with non-pathogenic one. Since, as it was found an *E. coli* UM146 was an intracellular strain in confrontation with the cell line and *E. coli* UM147 was an example of attaching to the HT-29 cells variant type pathogen, different pools of genes were expressed after 3h incubation time for these two pathogenic strains. Only one common gene transcript CCDC65 with identical RNA quantity in HT-29 cells after incubation with three *E. coli* strains tested, was identified (Table 1). CCDC65 plays a critical role in ciliary and flagellar motility driven by the nexin-dynein regulatory complex (N-DRC) as it has been demonstrated using defective gene mutations study by Bower *et al*. 4. REACTOME analysis revealed that only 6 RNA transcripts among 78 identified participated in 16 biochemical pathways for *E. coli* UM146 treated monolayer adenocarcinoma cells. In the case of *E. coli* UM147 16 RNA transcripts among 81 detected were involved in 50 biochemical pathways presented in Table 2A. Platelet aggregation initiated by *E. coli* UM146 suggests risk of heart failure in the case of human infection. Proteins involved in this process RAP1A and ITGB3 participate in 24 out of 27 possible biochemical reactions of plug formation known in REACTOME database. In addition, the same two proteins RAP1A and ITGB3 are involved in all 24 possible bioreactions during the response of HT-29 monolayer cells to bacterial invasion stimulated by integrins (Table 2A). Phospholipid metabolism regulated by PIKFYVE, SLC44A2, MTMR14 and HADH proteins was initiated in concordance with beta-oxidation processes of phospholipids fatty acids after incubation of HT-29 adenocarcinoma cells with *E. coli* UM147 (Table 2B). Also, several interleukins including interleukins 2, 3, 5, 7, 9, 15, 21 involved in signaling processes of plausible infection progress were activated during this bacterial interaction with HT-29 cells, as indicated in Table 2B. In addition, autophagy process was initiated by *E. coli* UM147 as indicated by expression of mRNA transcripts for RAB40B, ATG16L2, MTMR14 and MLST8 proteins what means that phagocytosis process was initiated by HT-29 cells.

**Figure 1:**
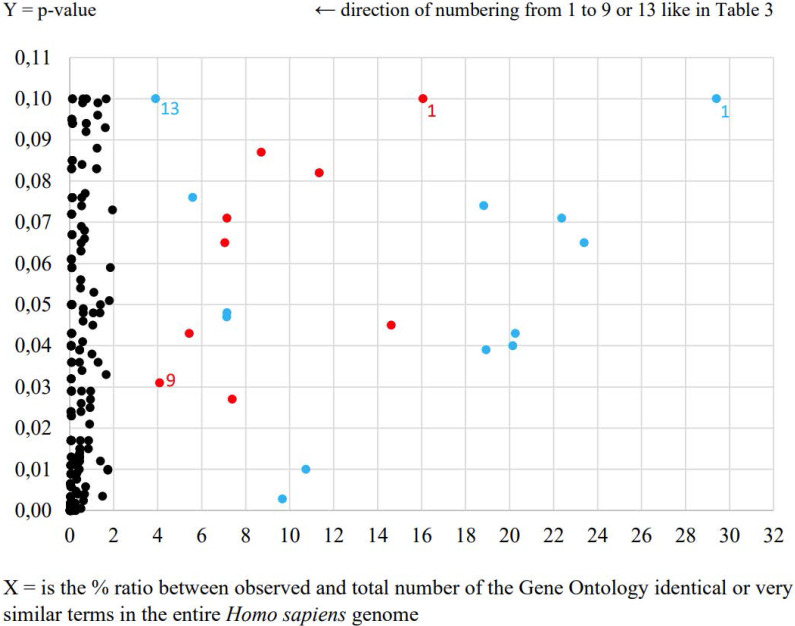
The most relevant Gene Ontology terms including Processes, Functions and Cellular Component in the response to *E. coli* UM146 (red spots) and *E. coli* UM147 (blue spots).

During the second data-mining analysis by agriGO online tool very intriguing negative regulation of adaptive immune response process was determined in the case of *E. coli* UM147 strain presented in Table 3 and Figure 1. Obtained result is an example of more precise in our opinion characterization of signaling interleukin processes of infection progress caused by this particular pathogenic strain in comparison to the results obtained from previous analysis. Furthermore, neural crest cell development process was also enriched in this analysis. What is interesting, the neural crest is a collection of multipotent stem cells that helps to develop the autonomic nervous system and is characterised by multipotency providing possible future applications in regenerative medicine 5. In the case of other pathogenic *E. coli* strain only general processes like positive regulation of transcription from RNA polymerase II promoter and positive regulation of RNA metabolic process were highlighted. Also, morphogenesis processes involved in HT-29 cell differentiation were significantly enriched (p<0.05) for *E. coli* UM146 strain (Table 3).

**Figure 2:**
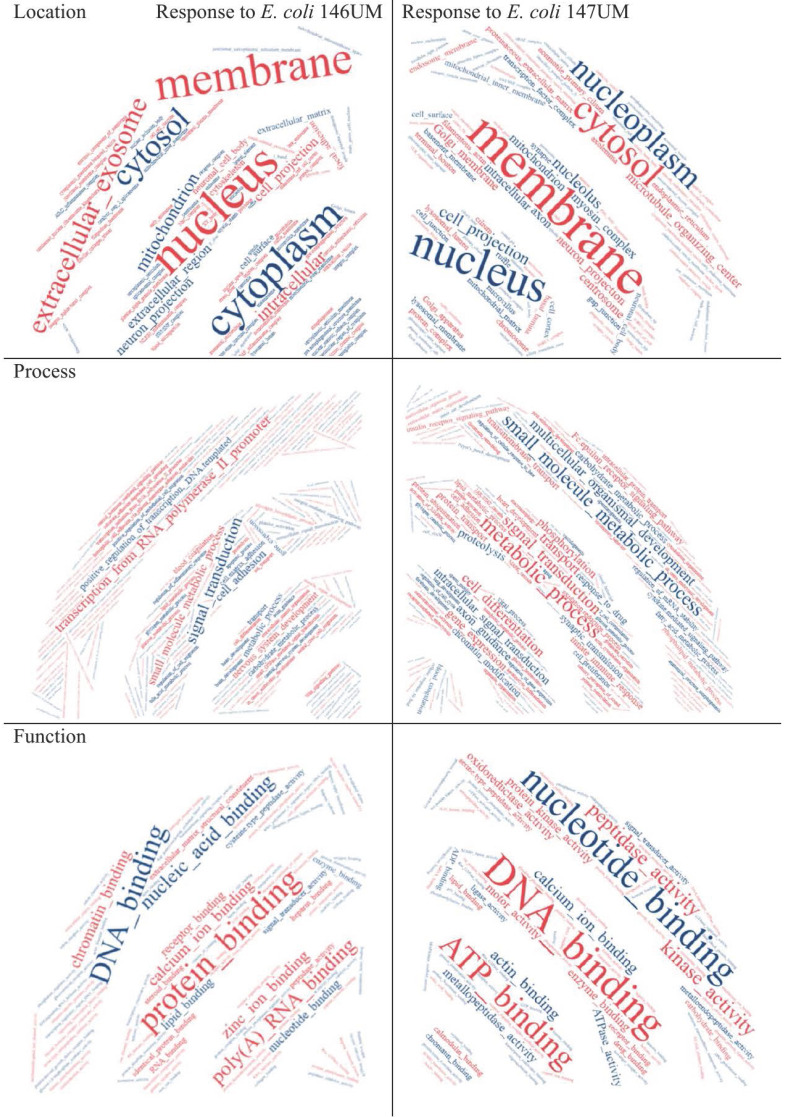
Ontology terms highlighted in word cloud application (https://www.wordclouds.com/) for all significant (p<0.1) results obtained from agriGO search.

Word cloud application results presented in Figure 2 provide general point of view on Gene Ontology terms predominantly enriched for Cellular Components what means location in HT-29 cells, Processes and Functions. Extracellular exosome was highlighted in location analysis what can be explained by trypsin-like activity of *E. coli* UM147 strain that is able to degrade intercellular connections in HT-29 monolayers. Some similarity in mode of action against Eucaryotic cells, can be seen in comparison to BFT toxins produced by enterotoxigenic *Bacteroides fragilis* strains 6, resulting in possible inflammation of the GI-tract 7, 8. Transcription from RNA polymerase II promoter was highlighted among Processes for *E. coli* UM146 what is in agreement with the enrichment analysis from agriGO online tool applied. In the case of Functions, the most divergent parameter in the word-cloud analysis performed, peptidase and metallopeptidase activities were highlighted for *E. coli* UM147 what confirms previous conclusions about influence of trypsin-like activity on possible inflammation of the gastrointestinal tract in some IBD patients. Also, kinase activity was indicated as characteristic form *E. coli* UM 147 strain infection, what can suggest more active involvement of phosphorylation and/or dephosphorylation processes during protein-to-protein signalling regulation processes inside infected monolayer cells. For *E. coli* UM146 characteristic highlighted functions were however related to chromatin and poly(A) RNA molecules binding as well as calcium and zinc ion binding interactions usually occurring in the catalytic centers of enzymatic reactions more frequent in HT-29 cells during the course of UM146 *E. coli* infection model used.

From literature data about host-pathogen interactions using enteric bacteria we can highlight significant reduction of Campylobacter jejuni invasion and intracellular survival of HT-29 cell line after 4h incubation period with EcN followed by infection with C. jejuni 81-176 9. The nature of EcN inhibitory interaction is by stimulating the expression of genes of cell junction and intestinal barrier integrity. As a result, there is an increase of the HT-29 cells resistance to infection. In our model of host-pathogen interaction between pathogenic and non-pathogenic bacterial strains and HT-29 cell line were separately studied based on identical RNA transcripts responses from the same pull of genes. From our model however it is possible to recognise biochemical reactions where probiotic and pathogenic strains equally participate in the same processes, what can give better insight into therapeutic effect during medical treatment.

## Figures and Tables

**Table 1:** Equal HT-29 cell line RNA transcripts expression after 3h incubation with *E. coli* strains used

*E. coli* strains	RNA transcripts
*E. coli* UM146 and *E. coli* Nissle 1917, n=78	ARV1, ATXN3L, ATXN7L1, C10orf126, C11orf31, C18orf54, C20orf196, C21orf58, CCDC65, CEP162, CH507-42P11.6, DDC-AS1, DDX10, ECHDC3, FAM105A, FAM208B, FERMT2, FHL1, FOXP1, FRMD7, GALR3, H2BFXP, HBE1, ITGB3, JPH1, LINC00319, LINC00485, LOC100101478, LOC100287015, LOC101927268, LOC101928837, LOC102724851, LOC105374994, LOC286178, LOC440982, LOC613266, LOC643733, MICU1, MSRB3, NAA30, NCOA2, NCR1, NFAM1, NR2F1-AS1, NR3C1, NUDT17, NXPE3, OR52K3P, OVOL2, PBX4, PCDH12, PCDHB18P, PGM2L1, PGR, PIEZO1, PPP1R3C, PRELP, PWP2, RALGAPA2, RAP1A, REC8, S100G, SAMSN1, SERPINB1, SEZ6L, SLCO3A1, SPERT, SRSF1, SSFA2, THADA, TLE4, TNXB, UBXN8, UNC5C, WIPI2, ZEB2, ZIC1, ZNF775
*E. coli* UM147 and *E. coli* Nissle 1917, n=81	AGFG2, AGRN, ATG16L2, BATF2, C4orf47, CALB2, CALML3, CCDC65, CDRT1, CEACAM7, DECR2, E4F1, EHF, ENTPD1, ETV1, F11, FGF18, FMNL1, FOXA1, GPATCH4, GYG2, HADH, IFT140, IGHD3-16, LCP2, LGALS8, LINC00032, LOC100505841, LOC101927027, LOC101928020, LOC101928433, LOC105372733, LOC1720, LOXL2, MAPK8IP3, MARK1, MBTPS2, MED23, MLST8, MPEG1, MTMR14, MYO19, MYO3A, MYO6, NPHP4, NUP214, OGFRL1, OPLAH, PCDHAC2, PEBP1, PIAS1, PIKFYVE, PMPCA, POU2F3, PYGB, RAB40B, RALGDS, RHBDL1, RIPPLY3, RORC, RP9P, RTP3, SERBP1, SHCBP1L, SLC18A2, SLC1A2, SLC44A2, SMARCA4, SPAG8, STAT5A, THAP4, TLK1, TNP2, TOR3A, VEPH1, XPNPEP2, YY2, ZC3H7B, ZFP36L1, ZNF117, ZNF491

**Table 2:** REACTOME Significant (p<0.05) pathways detected in HT-29 cell line in the response to *E. coli* 146UM or *E. coli* 147UM infections identical with the control non-pathogenic *E. coli* Nissle 1917

Pathway identifier	Pathway name	Entities pValue	#Reactions found	#Reactions total	Mapped entities
**A. Response to *E. coli* UM146**					
R-HSA-383280	Nuclear Receptor transcription pathway	1,071743E-11	2	2	PGR;NR3C1
R-HSA-354194	GRB2: SOS provides linkage to MAPK signaling for Integrins	7,293580E-03	2	2	RAP1A;ITGB3
R-HSA-372708	p130Cas linkage to MAPK signaling for integrins	8,753419E-03	3	3	RAP1A;ITGB3
R-HSA-3322077	Glycogen synthesis	1,202797E-02	11	24	PPP1R3C;PGM2L1
R-HSA-354192	Integrin signaling	2,567075E-02	24	24	RAP1A;ITGB3
R-HSA-4090294	SUMOylation of intracellular receptors	2,689497E-02	2	25	PGR;NR3C1
R-HSA-8982491	Glycogen metabolism	3,070566E-02	12	39	PPP1R3C;PGM2L1
R-HSA-6802948	Signaling by high-kinase activity BRAF mutants	3,202079E-02	4	6	RAP1A;ITGB3
R-HSA-1251985	Nuclear signaling by ERBB4	3,609635E-02	2	34	PGR
R-HSA-5674135	MAP2K and MAPK activation	3,891867E-02	8	12	RAP1A;ITGB3
R-HSA-9656223	Signaling by RAF1 mutants	3,891867E-02	4	7	RAP1A;ITGB3
R-HSA-76009	Platelet Aggregation (Plug Formation)	4,480482E-02	24	27	RAP1A;ITGB3
R-HSA-6802955	Paradoxical activation of RAF signaling by kinase inactive BRAF	4,632490E-02	4	7	RAP1A;ITGB3
R-HSA-9649948	Signaling downstream of RAS mutants	4,632490E-02	4	7	RAP1A;ITGB3
R-HSA-6802946	Signaling by moderate kinase activity BRAF mutants	4,632490E-02	4	7	RAP1A;ITGB3
R-HSA-6802949	Signaling by RAS mutants	4,632490E-02	4	9	RAP1A;ITGB3
**B. Response to *E. coli* UM147**					
R-HSA-70221	Glycogen breakdown (glycogenolysis)	6,172902E-04	12	15	PYGB;GYG2
R-HSA-1266695	Interleukin-7 signaling	1,022987E-03	10	26	STAT5A;SMARCA4
R-HSA-9645135	STAT5 Activation	1,512084E-03	3	3	STAT5A
R-HSA-9027283	Erythropoietin activates STAT5	1,512084E-03	3	3	STAT5A
R-HSA-8949275	RUNX3 Regulates Immune Response and Cell Migration	1,859202E-03	2	5	RORC
R-HSA-8985947	Interleukin-9 signaling	2,240519E-03	4	13	STAT5A
R-HSA-8982491	Glycogen metabolism	2,586785E-03	21	39	PYGB;GYG2
R-HSA-9020958	Interleukin-21 signaling	2,655603E-03	2	5	STAT5A
R-HSA-2586552	Signaling by Leptin	3,104028E-03	5	19	STAT5A
R-HSA-9020558	Interleukin-2 signaling	3,585370E-03	5	19	STAT5A
R-HSA-8983432	Interleukin-15 signaling	4,645137E-03	5	17	STAT5A
R-HSA-77350	Beta oxidation of hexanoyl-CoA to butanoyl-CoA	5,831597E-03	2	4	HADH
R-HSA-77310	Beta oxidation of lauroyl-CoA to decanoyl-CoA-CoA	5,831597E-03	2	4	HADH
R-HSA-77348	Beta oxidation of octanoyl-CoA to hexanoyl-CoA	5,831597E-03	2	4	HADH
R-HSA-1170546	Prolactin receptor signaling	5,831597E-03	2	14	STAT5A
R-HSA-9702518	STAT5 activation downstream of FLT3 ITD mutants	7,841754E-03	13	14	STAT5A
R-HSA-77346	Beta oxidation of decanoyl-CoA to octanoyl-CoA-CoA	7,841754E-03	2	5	HADH
R-HSA-1839117	Signaling by cytosolic FGFR1 fusion mutants	9,330878E-03	1	14	STAT5A
R-HSA-2243919	Crosslinking of collagen fibrils	1,011898E-02	3	13	LOXL2
R-HSA-9725371	Nuclear events stimulated by ALK signaling in cancer	1,265287E-02	2	9	STAT5A
R-HSA-9670439	Signaling by phosphorylated juxtamembrane; extracellular and kinase domain KIT mutants	1,355277E-02	4	11	STAT5A
R-HSA-9669938	Signaling by KIT in disease	1,355277E-02	4	25	STAT5A
R-HSA-982772	Growth hormone receptor signaling	1,447970E-02	5	28	STAT5A
R-HSA-8854691	Interleukin-20 family signaling	1,447970E-02	3	56	STAT5A
R-HSA-1632852	Macroautophagy	1,488191E-02	10	87	RAB40B;ATG16L2; MTMR14;MLST8
R-HSA-1226099	Signaling by FGFR in disease	1,507210E-02	27	106	STAT5A;FGF18
R-HSA-9703648	Signaling by FLT3 ITD and TKD mutants	1,543329E-02	13	24	STAT5A
R-HSA-9703465	Signaling by FLT3 fusion proteins	1,741900E-02	8	18	STAT5A
R-HSA-9674555	Signaling by CSF3 (G-CSF)	2,058859E-02	9	21	STAT5A
R-HSA-9006335	Signaling by Erythropoietin	2,058859E-02	3	24	STAT5A
R-HSA-9612973	Autophagy	2,071701E-02	10	108	RAB40B;ATG16L2; MTMR14;MLST8
R-HSA-186763	Downstream signal transduction	2,282495E-02	1	16	STAT5A
R-HSA-5578998	Defective OPLAH causes OPLAHD	2,472363E-02	1	1	OPLAH
R-HSA-3878781	Glycogen storage disease type IV (GBE1)	2,472363E-02	1	1	GYG2
R-HSA-3858516	Glycogen storage disease type 0 (liver GYS2)	2,472363E-02	1	1	GYG2
R-HSA-1839124	FGFR1 mutant receptor activation	2,515676E-02	1	25	STAT5A
R-HSA-3232142	SUMOylation of ubiquitinylation proteins	3,009615E-02	2	3	NUP214;PIAS1
R-HSA-9682385	FLT3 signaling in disease	3,009615E-02	15	52	STAT5A
R-HSA-3249367	STAT6-mediated induction of chemokines	3,080947E-02	3	6	STAT5A
R-HSA-1566948	Elastic fibre formation	3,403180E-02	1	17	LOXL2
R-HSA-451927	Interleukin-2 family signaling	3,538602E-02	16	59	STAT5A
R-HSA-9607240	FLT3 Signaling	3,676091E-02	3	43	STAT5A
R-HSA-5655302	Signaling by FGFR1 in disease	3,815614E-02	1	35	STAT5A
R-HSA-512988	Interleukin-3; Interleukin-5 and GM-CSF signaling	3,957142E-02	5	38	STAT5A
R-HSA-1433557	Signaling by SCF-KIT	4,100645E-02	4	39	STAT5A
R-HSA-9673221	Defective F9 activation	4,286870E-02	1	1	F11
R-HSA-77286	mitochondrial fatty acid beta-oxidation of saturated fatty acids	4,393460E-02	15	29	HADH
R-HSA-1483191	Synthesis of PC	4,693824E-02	1	18	SLC44A2
R-HSA-1483257	Phospholipid metabolism	4,814536E-02	11	218	PIKFYVE;SLC44A2; MTMR14;HADH

1: **Pathway identifier**; 2: **Pathway name**; 3: **Reactions found**: The number of reactions in the pathway that are represented by at least one molecule in the submitted data set; 4: **Reactions Total**: The number of reactions in the specific pathway name that contain molecules found; 5: **Entities p-value**: The result of the statistical test for over-representation of RNA transcripts in reactions identified; 6: **Mapped entities**: RNA transcripts participating in pathways.

**Table 3:** AgriGO comparison of HT-29 cell line transcription responses to pathogenic *E. coli* strains UM146 and UM147 equal to the level of transcription of non-pathogenic strain *E. coli* Nissle 1917

No.	*E. coli* UM146 - red spots	*E. coli* UM147 - blue spots
1.	P: cellular component biogenesis	P: aminoglycan catabolic process
2.	**P: cellular component assembly**	P: regulation of bone remodeling
3.	P: nervous system development	P: negative regulation of immune system process
4.	P: positive regulation of nucleobase, nucleoside, nucleotide and nucleic acid metabolic process	**P: regulation of phospholipase activity**
5.	**P: positive regulation of RNA metabolic process**	**P: embryonic organ morphogenesis**
6.	P: positive regulation of transcription	**P: positive regulation of biosynthetic process**
7.	P: positive regulation of transcription, DNA-dependent	P: amine metabolic process
8.	**P: positive regulation of transcription from RNA polymerase II promoter**	**P: negative regulation of adaptive immune response**
9.	**P: cell morphogenesis involved in differentiation**	**P: neural crest cell development**
10.	Less than 3 RNA transcripts involved	**P: regulation of biological process**
11.		**P: regulation of protein transport**
12.		P: oligodendrocyte differentiation
13.		P: steroid biosynthetic process

Bold font style indicates significant processes at p-value <0.05, while normal fonts p-value <0.1.
